# Characteristic findings of appendicular endometriosis treated with single incision laparoscopic ileocolectomy: Case report

**DOI:** 10.1016/j.ijscr.2019.12.039

**Published:** 2020-01-09

**Authors:** Keishi Hakoda, Masanori Yoshimitsu, Masashi Miguchi, Toshihiko Kohashi, Hiroyuki Egi, Hideki Ohdan, Naoki Hirabayashi

**Affiliations:** a Department of Gastroenterological and Transplant Surgery, Applied Life Sciences, Institute of Biomedical & Health Sciences, Hiroshima University, Kasumi 1-2-3 Minami-ku, Hiroshima-shi, Hiroshima, Japan; b Department of Surgery, Hiroshima City Hiroshima Citizens Hospital, Motomachi 7-33 Naka-ku, Hiroshima-shi, Hiroshima, Japan; c Department of Surgery, Hiroshima Prefectural Hospital, Ujinakanda 1-5-54 Minami-ku, Hiroshima-shi, Hiroshima, Japan; d Department of Surgery, Hiroshima City Asa Citizens Hospital, Kabeminami 2-1-1 Asakita-ku, Hiroshima-shi, Hiroshima, Japan

**Keywords:** CT, computed tomography, BMI, body mass index, SILS, single-incision laparoscopic surgery, MLS, multi-port laparoscopic surgery, Endometriosis, Submucosal tumor, Colectomy

## Abstract

**Introduction:**

We report the case of characteristic findings of appendicular endometriosis that presented as a submucosal tumor in the cecum without any abdominal symptoms treated with single-incision laparoscopic ileocolectomy.

**Presentation of case:**

The patient was a 51-year-old woman (body mass index: 21.5) who underwent an examination to investigate the cause of a positive fecal occult blood test. Her laboratory test results were normal, without anemia or tumor marker elevation. Colonoscopy revealed a submucosal tumor in the cecum. Enhanced computed tomography (CT), fluorodeoxyglucose-positron emission tomography-CT and magnetic resonance imaging failed to produce a clear diagnosis. Given the malignant potentiality of the tumor, ileocolectomy was considered potentially necessary, but she wished for minimally invasive surgery. She ultimately underwent ileocolectomy and lymphadenectomy with single-incision laparoscopic surgery. A pathological examination revealed the lesion to be appendicular endometriosis, and 14 lymph nodes with no malignancy were resected.

**Conclusion:**

Appendicular endometriosis can present as a submucosal tumor in the cecum without any abdominal symptoms. Appendicular endometriosis should be considered in the differential diagnosis of ileocecal submucosal tumor. Single-incision laparoscopic ileocolectomy was useful procedure for cecum tumor resection.

## Highlights

• Appendicular endometriosis a rare entity which usually presents with symptoms of abdominal pain, melena, and constipation.
• Appendicular endometriosis can present as a submucosal tumor in the cecum without any abdominal symptoms.
• Single-incision laparoscopic surgery is an useful procedure for cecum tumor resection.

## 1 Introduction

Intestinal endometriosis is a rare entity that can cause abdominal pain, melena and bowel obstruction. In the literature, there are a number of reports of patients with appendicular endometriosis who presented with symptoms of likely acute appendicitis and were treated by appendectomy laparoscopically or laparotomically. The macroscopic findings of the lesion were often appendiceal wall-thickening and diagnosed as appendicular endometriosis with a pathological examination [[1], [2], [3], [4], [5], [6], [7], [8], [9], [10]].

Single-incision laparoscopic surgery (SILS) for colorectal disease is a relatively new technique and known to be a more difficult maneuver than multi-port laparoscopic surgery (MLS). Furthermore, the oncological outcomes remain unclear [11].

We herein report the characteristic findings of appendicular endometriosis that presented as a submucosal tumor in the cecum without any abdominal symptoms and was treated with single-incision laparoscopic ileocolectomy as an oncologically acceptable procedure. This work has been reported in line with the SCARE criteria [12].

## 2 Presentation of case

**Fig. 1:**
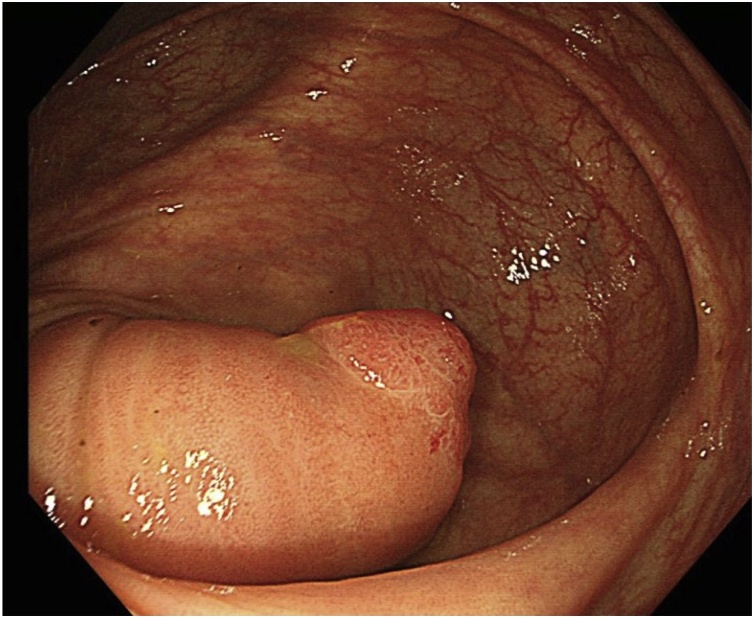
Colonoscopy showed a submucosal tumor in the cecum.

The patient was a 51-year-old woman whose body mass index was 21.5. She underwent an examination to determine the cause of a positive fecal occult blood test on cancer screening. She had no symptoms and she had no specific medical or family history. Her gynecological history was unknown. Her laboratory test results were normal, without anemia or tumor marker elevation. Colonoscopy showed submucosal tumor of 2.5 cm in diameter in the cecum (Fig. 1). Colonoscopic biopsy was not performed due to fear of complications. Enhanced computed tomography (CT) showed an enhanced tumor in the cecum. Magnetic resonance imaging revealed a cecal tumor with a low signal intensity on both T1- and T2-weighted imaging. There was no accumulation of fluorodeoxyglucose on positron emission tomography-CT, as a result, the preoperative diagnosis was unclear. The tumor was a potentially malignant lesion, so surgical resection was considered necessary, but she wished to receive minimally invasive surgery. Preoperatively, we had discussed and intended to perform a same surgical procedure as treating colon cancer. She ultimately underwent ileocolectomy and lymphadenectomy as SILS.

**Fig. 2:**
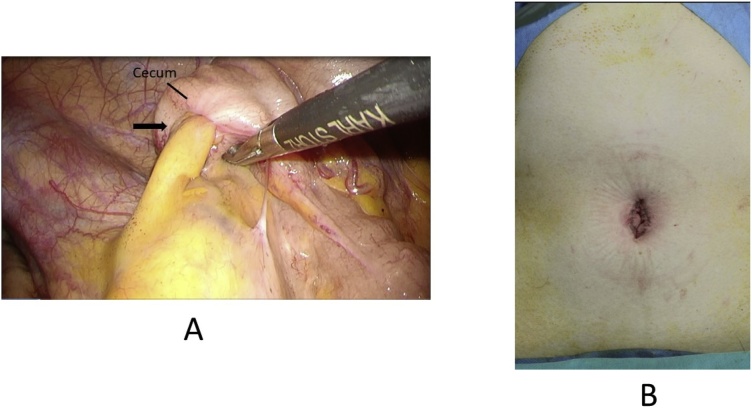
(A) A cecal tumor was observed as appendiceal intussusception to the cecum (arrow). (B) The suture wound: a vertical incision of approximately 4 cm in length was created through the umbilicus.

**Fig. 3:**
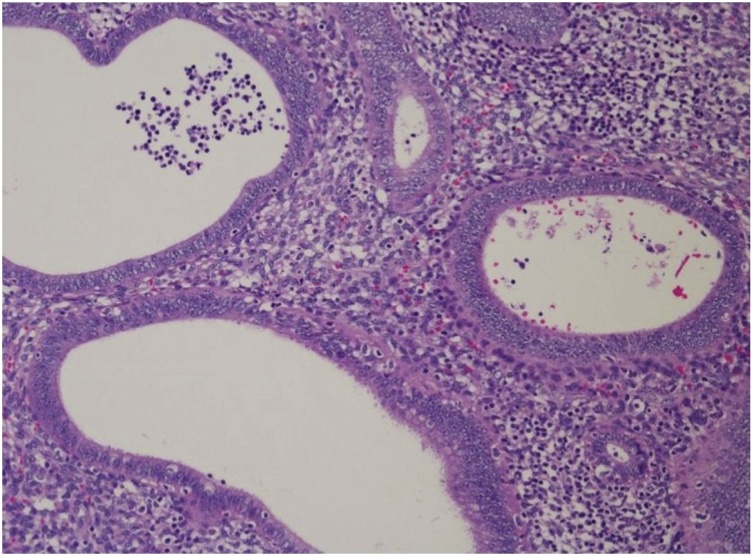
Hematoxylin and Eosin staining showed ectopic endometrial tissue on the appendix, which formed intussusception presenting as a submucosal tumor in the cecum.

The laparoscopic findings showed appendiceal intussusception to the cecum, which was considered a malignant entity during surgery (Fig. 2A). Although the impression differed from what was observed on preoperative imaging, we did not change the surgical procedure. We used GelPOINT® (AppliedMedical, Rancho Santa Margarita, CA, USA) for the surgery, which includes a Gelseal Cap, Alexis Wound Retractor, and 4 sleeves (ports). Initially, a 40-mm incision was placed at the umbilicus. The wound retractor accommodated the abdominal wall, and three sleeves (ports) were kept impaled on the Gelseal Cap. Pelvic exploration did not reveal ascites or any other signs of metastasis. Operator had expertise skills of MLS with some experience of SILS, then we performed ileocolic mobilization and lymphadenectomy with ileocolic artery and vein resection, similar to MLS. Functional end-to-end anastomosis was performed with a stapler (Echelon®60; Ethicon Endo-Surgery, Cincinnati, OH, USA) out of the body after removal via an umbilical incision (Fig. 2B). During the operation, there were no particular difficulties compared with MLS. The operation time and blood loss were 123 min and 15 ml, respectively. A pathological examination of the tumor showed ectopic endometrial tissue on the appendix, which had formed a mass and intussusception presenting as a submucosal tumor in the cecum; appendicular endometriosis was diagnosed based on these findings (Fig. 3). At 6 days after the operation, the patient was discharged without any complications and has been followed up in our hospital.

## 3 Discussion

Endometriosis is defined as an ectopic occurrence of tissue that functionally resembles endometrial tissue, which is implanted into regions other than the uterus [1]. It is thought to initially manifest as bleeding, which is followed by inflammation, which activates fibrin deposition, adhesion formation, and eventually, the scarring and distortion of the peritoneal surfaces of the organs and pelvic anatomy [2]. Appendicular endometriosis is a rare entity found in roughly 3% of cases of intestinal endometriosis and presents with symptoms of abdominal pain, melena, and constipation [3,4]. Approximately half of endometriosis patients suffer symptoms that are related to their menstrual cycle [3]. A number of previous reports have described the clinical course of appendicular endometriosis, noting that such cases usually present with abdominal pain, similar to acute appendicitis, and are then treated by appendectomy in an emergency setting and only postoperatively diagnosed as appendicular endometriosis based on pathological findings [[6], [7], [8], [9], [10]]. However, in the present case, the patient had no abdominal pain and no menstruation-associated symptoms, and the lesion manifested as a submucosal tumor 2.5 cm in diameter in the cecum.

When an ileocecal submucosal tumor is detected, the following differential diagnoses should be considered: adenocarcinoma, carcinoid, mucinous cystadenoma, mucinous cystadenocarcinoma, lipoma, lymphoma, and pericolonic abscess [13]. Considering its malignant potential, surgical resection should be performed, but the surgical procedure may be discussed, including the need for lymphadenectomy and the bowel resection range. Considering the present patient’s wish and the oncological curability, she ultimately underwent ileocolectomy and lymphadenectomy as SILS. As a result, the lesion was diagnosed as benign, but 14 lymph nodes were resected. Considering the number of resected lymph nodes, accomplished surgery was acceptable procedure even if the lesion was malignant.

SILS is a less invasive surgery than MLS, but it is known to be a difficult maneuver, and the oncological outcomes are unclear [11]. During surgery, as in previous reports about appendicular endometriosis, we did not observe any adhesion or inflammatory reaction around the cecum. Considering the natural course of the inflammation of endometriosis, her lesion might be in the early phase; in addition, the patient was a thin female with no history of abdominal surgery; these conditions allowed her to successfully undergo a low-invasive, cosmetically beneficial, and oncologically acceptable procedure without any particular difficulties during the operation.

The preoperative diagnosis of ileocecal submucosal tumors is difficult because these entities do not have specific imaging patterns. In the present case, we therefore performed ileocolectomy and lymphadenectomy to remove a benign tumor. Considering the difficulty of making a preoperative diagnosis, the surgical procedure performed in this patient might be appropriate in this case; however, the present case shows that endometriosis should also be considered in the differential diagnosis of ileocecal submucosal tumor, and further reports should be accumulated to determine preoperative diagnosis more clearly.

## 4 Conclusions

We reported a case of appendicular endometriosis that presented as a submucosal tumor in the cecum without any abdominal symptoms and which was successfully treated by single-incision laparoscopic ileocolectomy. Appendicular endometriosis should be considered in the differential diagnosis of ileocecal submucosal tumor.

## Units

International System of Units (SI).

## Sources of funding

The authors declare that no sources of funding have been requested for this research.

## Ethical approval

This case report is not research study, therefore approval was not given.

The ethical approval has been exempted by our institution.

## Consent

Written informed consent was obtained from the patient for publication of this case report and accompanying images. A copy of the written consent is available for review by the Editor-in-Chief of this journal on request.

## Author contribution

MY contributed to conceptualization, data curation, writing original draft and editing. MM, TK, HE, HO and NH contributed to supervision, writing original draft and editing.

## Registration of research studies

This paper is a clinical report, so the authors declare that no registration is needed.

## Guarantor

Masanori Yoshimitsu is the Guarantor for this work.

## Provenance and peer review

Not commissioned, externally peer-reviewed.

## Declaration of Competing Interest

The authors declare that have no conflicts of interest.
